# Distinct Firing Activities of the Hypothalamic Arcuate Nucleus Neurons to Appetite Hormones

**DOI:** 10.3390/ijms23052609

**Published:** 2022-02-26

**Authors:** Junewoo Na, Byong Seo Park, Doohyeong Jang, Donggue Kim, Thai Hien Tu, Youngjae Ryu, Chang Man Ha, Marco Koch, Sungchil Yang, Jae Geun Kim, Sunggu Yang

**Affiliations:** 1 Department of Nano-Bioengineering, Incheon National University, Incheon 406-772, Korea; nace3333@gmail.com (J.N.); longheadbrother@gmail.com (D.J.); donggue1209@gmail.com (D.K.); 2 Division of Life Sciences, College of Life Sciences and Bioengineering, Incheon National University, Incheon 406-772, Korea; 2021s135@inu.ac.kr (B.S.P.); thaihientu@gmail.com (T.H.T.); 3 Research Division and Brain Research Core Facilities of Korea Brain Research Institute, Daegu 41068, Korea; ryj123@kbri.re.kr (Y.R.); changman@kbri.re.kr (C.M.H.); 4 Anatomy and Cellular Biology, Institute of Theoretical Medicine, Medical Faculty, University of Augsburg, Universitätsstrasse 2, 86159 Augsburg, Germany; marco.koch@med.uni-augsburg.de; 5 Institute of Anatomy, Medical Faculty, University of Leipzig, Liebigstr. 13, 04103 Leipzig, Germany; 6 Department of Neuroscience, City University of Hong Kong, Tat Chee Avenue, Kowloon, Hong Kong SAR, China; sungchil.yang@cityu.edu.hk

**Keywords:** AgRP, POMC, dopamine, ghrelin, leptin, appetite

## Abstract

The hypothalamic arcuate nucleus (Arc) is a central unit that controls the appetite through the integration of metabolic, hormonal, and neuronal afferent inputs. Agouti-related protein (AgRP), proopiomelanocortin (POMC), and dopaminergic neurons in the Arc differentially regulate feeding behaviors in response to hunger, satiety, and appetite, respectively. At the time of writing, the anatomical and electrophysiological characterization of these three neurons has not yet been intensively explored. Here, we interrogated the overall characterization of AgRP, POMC, and dopaminergic neurons using genetic mouse models, immunohistochemistry, and whole-cell patch recordings. We identified the distinct geographical location and intrinsic properties of each neuron in the Arc with the transgenic lines labelled with cell-specific reporter proteins. Moreover, AgRP, POMC, and dopaminergic neurons had different firing activities to ghrelin and leptin treatments. Ghrelin led to the increased firing rate of dopaminergic and AgRP neurons, and the decreased firing rate of POMC. In sharp contrast, leptin resulted in the decreased firing rate of AgRP neurons and the increased firing rate of POMC neurons, while it did not change the firing rate of dopaminergic neurons in Arc. These findings demonstrate the anatomical and physiological uniqueness of three hypothalamic Arc neurons to appetite control.

## 1. Introduction

The hypothalamus is a master unit that regulates energy homeostasis through the cross-correlated operation of endocrine and sympathetic nervous systems. The hypothalamus consists of various nuclei that are mutually interconnected for efficient energy metabolisms, such as the ventromedial nucleus of the hypothalamus (VMH), the dorsomedial nucleus of the hypothalamus (DMH), lateral hypothalamus (LH), and arcuate nucleus (Arc). Among them, the Arc dynamically regulates energy intake and expenditure via the on-demand integration of afferent inputs, such as circulating nutrients, hormones, and neurotransmitters [1,2]. The circuit activity of the hypothalamic Arc can be mainly executed by two distinct neuronal populations of satiety-triggering POMC neurons and hunger-promoting AgRP neurons [3,4]. As such, POMC and AgRP neurons have significantly increased the firing rate upon leptin and ghrelin treatment, respectively [5,6,7].

Studies for the hypothalamic dopaminergic neurons were initiated with the findings showing their projection to the median eminence where axon terminals release dopamine into the hypophyseal portal system for inhibiting prolactin secretion from the anterior pituitary gland [8]. Although recent studies have identified that dopaminergic neurons are also present in the dorsal part of the hypothalamic Arc [9] and their excitability responds to the starvation and ghrelin treatment [9], their functional roles in the regulation of energy metabolism have not been completely unraveled. For now, further studies are required for clarifying the anatomical and electrophysiological characterizations of these three neurons in the regulation of appetite. Here, we determined the precise spatial distribution of the three neurons of the hypothalamic Arc by using 3D reconstructed images. Furthermore, we investigated the cell type-specific intrinsic properties of AgRP, POMC and dopaminergic neurons, as well as their responsiveness to ghrelin, an orexigenic hormone, and leptin (an anorexigenic hormone), by using whole-cell patch-clamp recordings. For these experiments, we utilized genetically engineered mouse lines that are individually tagged with fluorescence reporter proteins to POMC, AgRP, or dopamine active transporter (DAT)-positive neurons. The current study unveiled the distinct firing properties of each Arc neuron to appetite hormones in addition to its geographical organization, thereby revealing how Arc neurons regulate the energy metabolism for appetite control. 

## 2. Results

### 2.1. Three-Dimensional Distribution of AgRP, POMC, and TH-Positive Neurons in the Hypothalamic Arc

**Figure 1:**
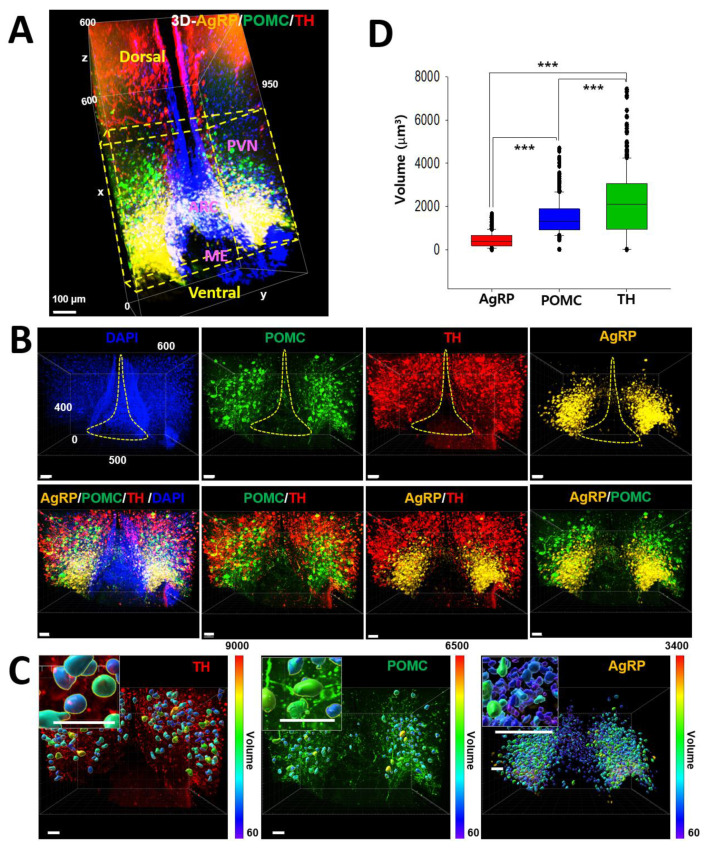
Distribution of POMC, AgRP, and TH-positive neurons in the hypothalamic arcuate nucleus. The hypothalamic Arc of the mouse brain was selected to determine the 3D distribution of POMC, AgRP, and TH-positive neurons. (**A**). The yellow dotted box showed the concurrent region of three POMC (green), AgRP (yellow), and TH-positive cells (red). AgRP, agouti-related protein; POMC, proopiomelanocortin; TH, tyrosine hydroxylase; PVN, paraventricular nucleus; Arc, arcuate nucleus; ME, median eminence. X axis, 950 µm; Y axis, 600 µm; Z axis, 600 µm. (**B**). The co-localization of POMC, AgRP, and TH-positive neurons in the yellow Arc region of X-axis, 400 µm; Y axis, 500 µm; Z-axis, 600 µm. In the second row, dual-labeled images showed that none of these neurons overlapped in the hypothalamic Arc. Scale bars: 100 µm. The yellow dot guideline shows the third ventricle area. (**C**). Representative 3D images of cell bodies using the Imaris software 9.2. Insets showed examples of the high magnification area for soma volume analysis. Inset scale bars: 50 µm. (**D**). Significant differences in the soma volume were observed in TH, POMC, and AgRP in descending order. The results were presented as the means ± SEMs. *** *p* < 0.001.

**Figure 2:**
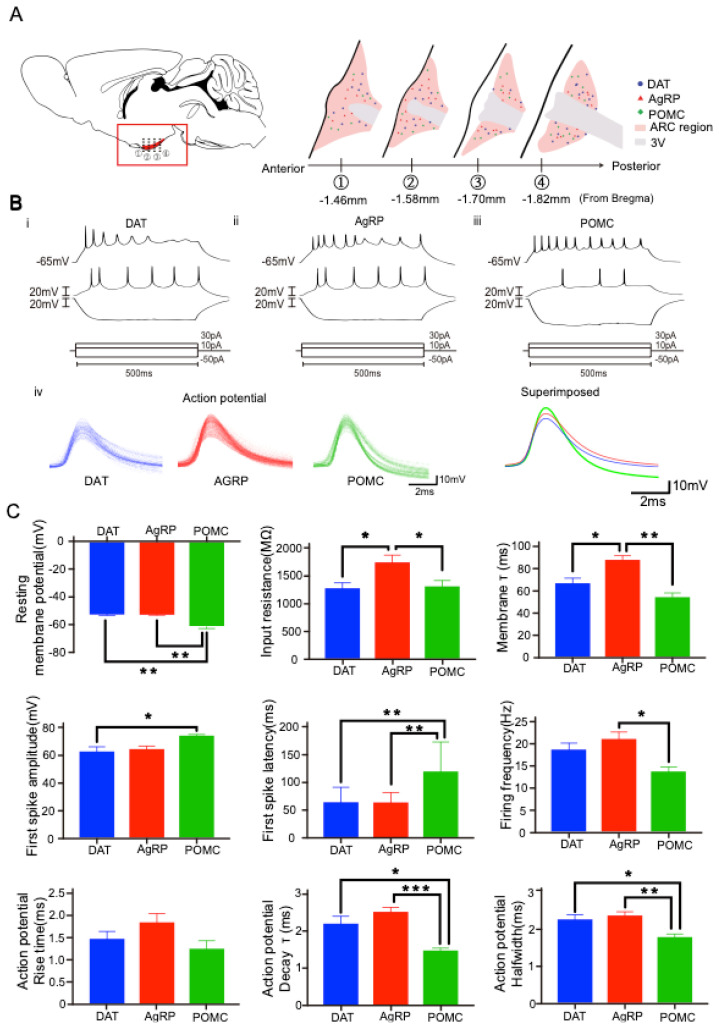
Intrinsic properties of individual Arc neurons. Distinct electrophysiological properties of each Arc neuron. (**A**). Whole-cell patch-clamp recordings were carried out for identifying the firing and intrinsic properties of DAT-positive, AgRP, and POMC neurons in the marked location. The thick black line was a border of the ventricular region and the pink area was the Arc region. The blue circle, red triangle, and green diamond dots indicated DAT-positive, AgRP, and POMC neurons, respectively. (**B**). Responses to long step currents (at 30, 20 pA, and −50 pA during 500 ms) were shown in DAT-positive, AgRP, and POMC neurons. The response was measured under the current-clamp mode with the RMP of −65 mV. (**C**). Each Arc neuron showed unique intrinsic membrane properties of RMP, input resistance, membrane tau, first spike amplitude, first spike latency, firing frequency, action potential rise time, action potential decay tau, and action potential halfwidth of Arc neurons. The results were presented as the means ± SEMs. * *p* < 0.05, ** *p* < 0.01, *** *p* < 0.001.

To identify the distribution of AgRP, POMC, and tyrosine hydroxylase (TH)-positive dopaminergic neurons in the hypothalamic Arc, we obtained three-dimensional (3D) images of AgRP (yellow), POMC (green), and TH (red) (Figure 1A). AgRP neurons were largely located in the proximal to the third ventricle or median eminence (ME), while POMC neurons were mostly distributed in the distal to the third ventricle (i.e., the lateral part of the Arc). TH-positive neurons were abundantly found in the dorsal part of the Arc. We further examined whether there is co-localization among AgRP, POMC, and dopaminergic neurons with high magnification of images. Intriguingly, dual-labeled images showed that none of these neurons overlapped in the hypothalamic Arc (Figure 1B). We additionally evaluated the volume of the cell body with high-resolution images and found the labelled size of TH, POMC, and AgRP in descending order (Figure 1C,D). Although the soma volume was measured with the images labeled with each molecular marker, the calculated volume appears to correspond to the intrinsic properties of each neuron, as shown in Figure 2. The fact that each neuron is structurally situated in different areas of the hypothalamic Arc suggests location-dependent segregation of cellular functions.

### 2.2. Distinct Electrophysiological Properties of Each Arc Neuron

Whole-cell patch-clamp recordings were carried out for identifying the firing and intrinsic properties of AgRP, POMC, and dopamine transporter (DAT)-positive neurons that are mostly located surrounding the third ventricle (Figure 2A). Each neuron showed different response patterns to long step-current injection (10 pA steps from −50 pA to 30 pA with 500 ms duration). The spiking of DAT-positive and AgRP neurons was quickly adapted, unlike that of POMC neurons (Figure 2B). The intrinsic membrane properties were scrutinized. POMC neurons showed significantly lower resting membrane potential (RMP) than the other two neurons (F (2, 56) = 15.28, *p* < 0.0001) (Figure 2C). AgRP neurons showed significantly higher input resistance than the other neurons without a difference between DAT-positive and POMC neurons (F (2, 52) = 5.812, *p* = 0.0053). AgRP neurons also showed significantly longer tau (τ) values than the other neurons without a significant difference between DAT-positive and POMC neurons (F (2, 49) = 11.23, *p* < 0.0001). As shown in Figure 1, the smallest cell size of AgRP neurons may reflect the highest input resistance and the longest tau among the other neurons. POMC neurons showed a significantly higher first spike amplitude than the other neurons without a significant difference between DAT and AgRP neurons (F (2, 56) = 3.231, *p* = 0.0470). POMC neurons showed significantly longer first spike latency than the other neurons without a significant difference between DAT-positive and AgRP neurons (F (2, 48) = 14.23, *p* < 0.0001). POMC neurons showed significantly lower firing frequency than DAT-positive and AgRP neurons without a significant difference between DAT-positive and AgRP neurons (F (2, 50) = 4.600, *p* = 0.0147). There was no significant difference among three groups in rise time of action potentials (Aps) (F (2, 30) = 2.320, *p* = 0.116; POMC vs. AgRP: *p* =0.105; POMC vs. DAT: *p* = 0.735; DAT vs. AgRP: *p* = 0.401). The constant decay time of APs of POMC neurons was significantly lower than AgRP and DAT-positive neurons with no in-between difference (F (2, 30) = 11.596, *p* < 0.001; POMC vs. AgRP: *p* < 0.001; POMC vs. DAT: *p* < 0.05; DAT vs. AgRP: *p* = 0.325). POMC neurons had narrower halfwidth of APs than DAT-positive and AgRP neurons with no in-between difference (F (2, 30) = 7.337, *p* < 0.05; POMC vs. AgRP: *p* < 0.01; POMC vs. DAT: *p* < 0.05; DAT vs. AgRP: *p* = 0.794). Given the unique intrinsic membrane and firing properties of each Arc neuron, we may identify a characteristic neuron even without the anatomical visualization of a target neuron.

### 2.3. Heterogeneous Firing Patterns of Arc Neurons Depending on the RMP

**Figure 3:**
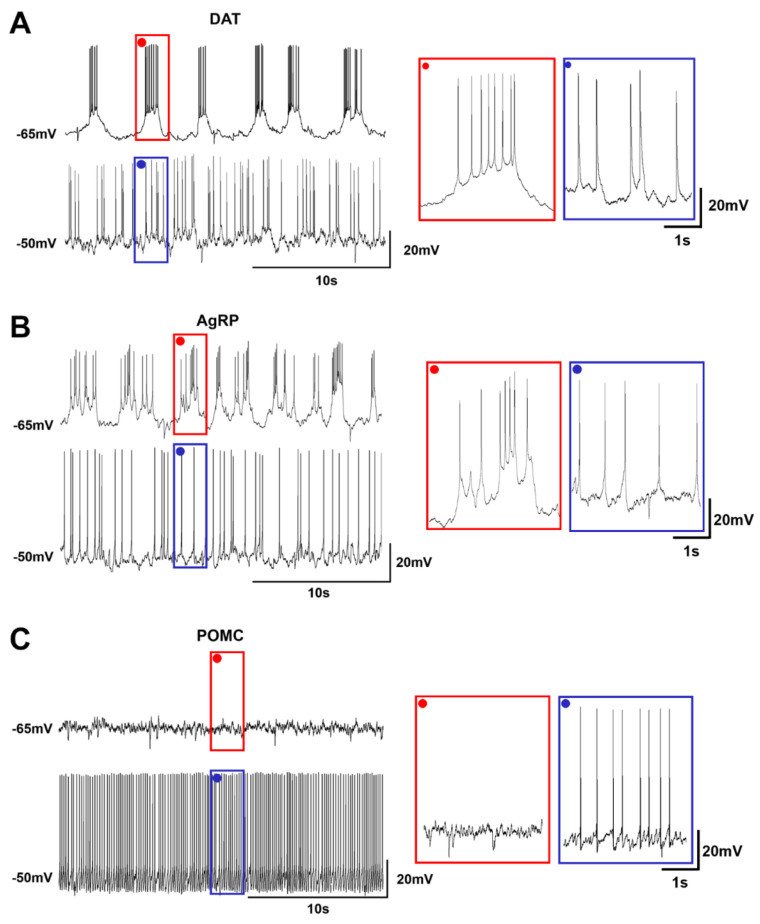
Firing patterns of Arc individual neurons. Heterogeneous firing patterns of Arc neurons depending on RMPs. Red box indicates the enlarged firing pattern of each neuron at −65 mV. Blue box indicates the enlarged firing pattern of each neuron at −50 mV. (**A**). The firing pattern of DAT neurons was a burst mode at −65 mV and a tonic mode at −50 mV. (**B**). The firing pattern of AgRP neurons was a burst mode at −65 mV and a tonic mode at −50 mV. (**C**). The firing pattern of POMC neurons had no firing at −65 mV and became a tonic mode at −50 mV.

Each Arc neuron had a specific firing pattern depending on the RMP. DAT-positive neurons showed a regular burst firing pattern at −65 mV, while a tonic firing pattern appeared when the resting potential was depolarized to −50 mV (Figure 3A). Similarly, the firing pattern of AgRP neurons showed bursting, albeit being irregular, and was converted into a tonic firing pattern at −50 mV (Figure 3B). In sharp contrast, POMC neurons did not possess burst firing at any potential levels, and only had a tonic firing pattern when depolarized (Figure 3C). This observation suggests that all Arc neurons promote the sustained release of endocrines when activated, while DAT-positive and AgRP neurons induce an abrupt release of endocrine at rest.

### 2.4. Ghrelin and Leptin Reactivity of the Arc Neurons

**Figure 4:**
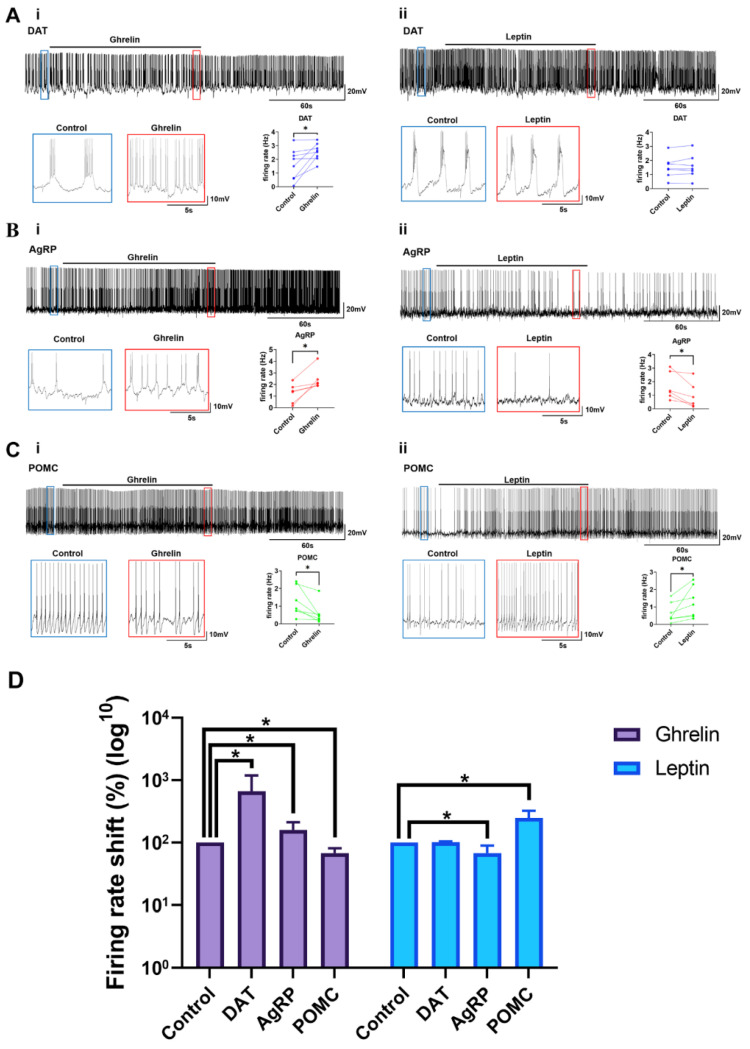
Reactivity of Arc neurons to ghrelin and leptin. Distinct responses of the hypothalamic Arc neurons to ghrelin and leptin. (**Ai**). DAT-positive neurons showed a significantly increased firing rate when ghrelin was applied. (**Aii**). There was no significant difference between before and after leptin. (**Bi**). AgRP neurons showed a significantly increased firing rate when ghrelin was applied. (**Bii**). AgRP neurons showed a decreased firing rate when leptin was applied. (**Ci**). POMC neurons showed a decreased firing rate when ghrelin was applied. (**Cii**). POMC neurons showed a significantly increased firing rate when leptin has applied. (**D**). A summary of the firing rate alteration upon ghrelin and leptin. The results were presented as the means ± SEMs. * *p* < 0.05.

The hypothalamic Arc regulates the whole-body energy metabolism by integrating the multiple factors that reflect the status of energy availability, such as nutrients, hormones, and neuronal inputs. Ghrelin, a gut-derived hormone, triggers a hunger signal, and leptin, an adipose-derived hormone, enhances a satiety signal via targeting the hypothalamic neurons involved in appetite regulation. We interrogated the activity patterns of AgRP, POMC, and DAT-positive neurons in response to bath application of ghrelin or leptin. The firing rate of DAT-positive neurons was significantly increased during ghrelin application. Upon ghrelin, the membrane potential was depolarized and, accordingly, the firing pattern was changed from a burst to tonic mode (*n* = 8, *p* = 0.0251) (Figure 4(Ai)). In sharp contrast, leptin did not alter the resting membrane potential and firing pattern in DAT-positive neurons (*n* = 7, *p* = 0.4722) (Figure 4(Aii)). The firing rate of AgRP neurons was significantly increased with ghrelin (*n* = 6, *p* = 0.0180) (Figure 4(Bi)), but decreased upon leptin (*n* = 6, *p* = 0.0205) (Figure 4(Bii)). As opposed to the effects on AgRP neurons in response to ghrelin or leptin treatments, the firing rate of POMC neurons was decreased and increased upon ghrelin (*n* = 7, *p* = 0.0314) (Figure 4(Ci)) and leptin (*n* = 7, *p* = 0.0478) (Figure 4(Cii)), respectively. These results confirm an opposite response between AgRP and POMC neurons in light of neuronal activities in response to ghrelin and leptin, and newly demonstrate that DAT-positive neurons in the Arc respond to ghrelin, but not to leptin.

## 3. Discussion

Previous studies on POMC, AgRP, and dopaminergic neurons in the hypothalamic Arc focused on their roles in energy metabolism and on their interactions in the context of hormonal regulation [9,10,11,12,13]. Here, our study newly demonstrated the distinct anatomical and electrophysiological properties of three Arc neurons and their susceptibility to appetite hormones. We found these neurons’ anatomical and biophysical segregation in the Arc. Furthermore, we denoted that these three neurons have distinct firing patterns and differential responses to ghrelin and leptin. 

AgRP neurons have irregularly tonic spikes to the stimulus when they are depolarized [14,15,16]. Moreover, according to our findings of higher input resistance/longer time constant and smaller AgRP-labelled soma size, our study confirms a previous study that AgRP neurons can have smaller cell sizes than the other Arc neurons [16]. Together with the higher input resistance and estimated small cell size, AgRP neurons with wider half-width and slower decay τ of APs may reflect fewer voltage-gated K^+^ channels than the other Arc neurons. The firing pattern of AgRP neurons transforms from a burst to tonic mode when hyperpolarized, suggesting differential modulation of food metabolism which heavily relies on a resting membrane state. POMC neurons have more hyperpolarized RMP, lower input resistance, and slower time constant than the other Arc neurons. These intrinsic membrane properties likely correspond to the longer decay τ and narrower half-width of spikes. The hyperpolarized RMP of POMC neurons may be caused by an intervention of gamma-aminobutyric acid (GABA) receptors [17]. POMC neurons heavily receive GABAergic inputs (in particular, tonic GABA) from AgRP neurons, resulting in the hyperpolarization of POMC neurons and making them quiescent at the RMP [18,19]. Due to the lack of burst firing in POMC neurons, they are likely to be involved in the tonic release of neurotransmitters irrespective of the membrane potentials [20]. DAT-positive neurons have a lower input resistance and slower membrane time constant than AgRP neurons. DAT-positive neurons’ intrinsic characteristics correspond to a large cell size when soma volume is measured with TH-staining immunohistochemistry. This result leads us to speculate that DAT-positive neurons have numerous K^+^ channels and larger cell sizes than the other Arc neurons as the number of K^+^ channels is closely related to the input resistance and the time constant of neurons [14,15,21,22]. Another noticeable finding for DAT-positive neurons is a bursting firing pattern when hyperpolarized. Previous studies have documented that a burst firing is highly related to various cellular functions, such as enhanced neuroplasticity, signal filtering, or synchronization, which suggests the essential roles of DAT-positive neurons in the regulation of neuroendocrine release [23,24,25].

Orexigenic AgRP neurons show a biphasic effect in response to two appetite hormones, i.e., ghrelin and leptin. Ghrelin induces the burst spiking while leptin suppresses the spiking. As the bursting promotes synaptic release [26,27], ghrelin may increase neurotransmitter release in the AgRP neuron. These results are consistent with the previous well-known findings in which AgRP neurons are activated and inhibited upon ghrelin and leptin, respectively [2,6]. As calcium-permitting channels (such as NMDA receptors and voltage-gated Ca^2+^ channels) and GABA_B_ receptors are also involved in the generation of the bursting firing pattern and the promotion of neurotransmitter release [28,29,30], further investigation is required for revealing the roles of calcium-permitting channels in the differential regulation of neuroendocrine release. Anorexigenic POMC neurons are activated by energy surfeits and inhibited by energy deficits. When activated, these neurons induce α-MSH release, which acts over the melanocortin-4 receptor (MC4R) located in different hypothalamic areas for increasing energy expenditure and reducing energy intake [3]. POMC neurons also show a biphasic effect in response to ghrelin and leptin, which is opposite to the responses of AgRP neurons. As previous studies have shown that ghrelin evokes direct excitatory effects to the dopaminergic neurons in the Arc [9], we successfully confirmed that Arc dopaminergic neurons have an increased firing rate in response to ghrelin treatment. Multiple lines of evidence have shown that leptin administration directly into the ventral tegmental area (VTA) leads to a reduction in food intake, while the knockdown of leptin receptors in the VTA results in an increase in food intake, locomotor activity, and hedonic feeding behaviors [31]. However, the dopaminergic neurons in the Arc do not respond to the anorexigenic hormone leptin. This suggests that dopaminergic neurons in Arc may have a preferential role in the regulation of the orexigenic signals. In accordance with firing patterns of dopaminergic neurons in the Arc, a previous study has shown that the optogenetic stimulation of Arc dopaminergic neurons leads to elevated feeding behavior via enhancing the activity of AgRP neurons and oppositely inhibiting the activity of POMC neurons [9]. Although the current study provides a useful information to better understand the functional roles of Arc dopaminergic neurons in the appetite regulation, further studies are required to clarify the complexity of the neural plasticity between arcuate neurons that controls whole-body energy metabolism. 

Collectively, the present study identifies the distinct histological, physiological and electrical properties of AgRP, POMC and dopaminergic neurons in the hypothalamic Arc, and suggests the functional segregation of arcuate neurons on the hypothalamic control of energy metabolism.

## 4. Materials and Methods

### 4.1. Animal Models

Transgenic mice (POMC-EGFP; stock no. 009593, AgRP-Cre stock no. 012899, DAT-Cre stock no. 006660, and Ai14 stock no. 007914) were obtained from Jackson Laboratory (Bar Harbor, ME, USA). The POMC-EGFP mice led to the expression of GFP in POMC neurons. To visualize AgRP-Cre or DAT-Cre expression, AgRP-Cre or DAT-Cre mice were crossed with Ai14 reporter mice to generate AgRP-Cre Ai14 or DAT-Cre Ai14 mice, while Cre expression can be directly visualized by tdTomato, a variant RFP, under fluorescent microscopes. These mice were used for patch-clamp recordings of the AgRP, POMC, and DAT neurons as described below. Genotyping for transgenic mice was confirmed using primers from Jackson Laboratory: POMC-EGFP, 5′-AAGTTCATCTGCACCACCG-3′ and 5′-TCCTTGAAGAAGATGGTGCG-3′ for 173 bp; AgRP-Cre, 5′-GCTTCTTCAATGCCTTTTGC-3′ and 5′-AGGAACTGCTTCCTTCACGA-3′ for 280 bp; DAT-Cre, 5′-TGGCTGTTGGTGTAAAGTGG-3′ and 5′-CCAAAAGACGGCAATATGGT-3′ for 152 bp; Ai14, 5′-GGCATTAAAGCAGCGTATCC-3′ and 5′-CTGTTCCTGTACGGCATGG-3′ for 196 bp. The mice were maintained in a temperature- (23–25 °C) and humidity-controlled chamber with a 12 h light–dark cycle (light exposure from 7 am to 7 pm). The mice had access to standard chow (D.B.L., Eumseong, Korea) and water ad libitum. All the animal care and experimental procedures were performed in accordance with the protocols approved by the Institutional Animal Care and Use Committee (IACUC) at the Incheon National University (permission number: INU-ANIM-2021-01). 

### 4.2. Sample Preparation of Hypothalamic Region for Deep Tissue Immunostaining

POMC-EGFP/AgRP Cre-tdTomato double-fluorescence-expressing mice were cardiac perfused with 4% PFA and the hypothalamus coronally cut to a thickness of 1 mm using the Leica VT100 S vibratome. The sections containing hypothalamic nuclei (stereotaxic coordinates: between −1.06 mm and −2.40 mm from the bregma) were matched with a mouse brain atlas book (Paxinos and Franklin, 2001, *The Mouse Brain in Stereotaxic Coordinates*—second edition, San Diego, CA, USA, Academic Press). The fixed brain slices were placed in the chamber of the C-phoresis (Crayon technologies, Guri-si, Gyeonggi-do, Korea) and followed the manufacture protocols. Briefly, brain slices were placed in the chamber with 15–20 mL of clearing buffer, and an electric field was applied with maintained 40 °C buffer. Then, 80 V of constant power was applied across the electrodes for 30 min for 1 mm-thick brain slices. The sample holder consisted of an acryl plate and porous nylon mesh. For immunostaining, the cleared brain slice was incubated for 2 h with blocking solution (10% Normal horse serum and 1% TritonX-100 in PBS (Sigma-Aldrich, St. Louis, MO, USA)) and washed on a gentle shaker twice for 30 min. Subsequently, mouse brain slices were stained using the electrophoretic immunostaining system (C-stain, Crayon technologies, Guri-si, Gyeonggi-do, Korea) with the antibody of rabbit anti-tyrosine hydroxylase (1/200, Cat# ab6211, Abcam, Cambridge, UK) for 2 h and washed for 30 min three times with a large volume of washing buffer. Washed brain slices were then processed in the C-stain system again with a goat anti-rabbit Alexa Fluor 647 (1/500, Cat# A-21245, Invitrogen, Carlsbad, CA, USA) for 2 h and washed for 30 min three times again. Washed brain slices were immersed with refractive index matching solution for 1 h and imaged with fast confocal microscopy (Dragonfly 502 w, Andor Technology, Belfast, UK). The objectives were a 10×, Plan Apochromat, and 20×, Apo LWD WI from Nikon. All acquired images were adjusted by Imaris software (ver 9.2.1, Bitplane, OXFORD Instruments, Abingdon, Oxfordshire, UK). 

### 4.3. Slice Preparation

The brain slices of hypothalamic arc region were obtained from POMC-EGFP, AgRP-Cre-Ai14, and DAT-Cre-Ai14 mice (postnatal age: 4–6 weeks). Mice were deeply anesthetized with isoflurane shortly before brain slicing. The brains were quickly shift into cold (4 °C), oxygenated (5% CO_2_, 95% O_2_) slicing medium containing 110 mM of choline chloride (Sigma-Aldrich), 2.5 mM of KCl (Sigma-Aldrich), 1.2 mM of NaH_2_PO_4_ (Sigma-Aldrich), 25 mM of NaHCO_3_ (Sigma-Aldrich), 20 mM of glucose (Sigma-Aldrich), 7 mM of MgCl_2_ (Sigma-Aldrich), and 0.5 mM of CaCl_2_ (Sigma-Aldrich). Coronal slices (150 μm) were cut using a vibratome. After slicing, the mice brain slices were transferred to a holding chamber filled with oxygenated (5% CO_2_, 95% O_2_) artificial CSF (ACSF) solution containing 124 mM of NaCl (Sigma-Aldrich), 2.5 mM of KCl, 1 mM of NaH_2_PO_4_, 26.2 mM of NaHCO_3_, 20 mM of glucose, 1.3 mM of MgCl_2_, and 2.5 mM of CaCl_2_. After at least one hour of recovery, individual slices were transferred to a recording chamber. Oxygenated ACSF continuously flew at a rate of 5 mL/min at 30–32 °C temperature. 

### 4.4. Patch-Clamp Recording 

The Axon clamp 700B amplifier and the Axon Digidata 1550A (Molecular Devices, San Jose, CA, USA) were used for whole-cell patch recording. The whole-cell patch-clamp application was performed on fluorescent neurons in the hypothalamic Arc region (AgRP-cre: red signal, POMC-EGFP: green signal, DAT-cre: red signal). BX51WI fluorescence microscope (Olympus, Tokyo, Japan) with a Zyla 5.5 CCD camera (Andor, Abingdon, UK) visualized the fluorescent Arc neurons. The recording pipettes tip resistance was 5–9 MΩ when filled with pipette internal solution containing 135 mM of K-gluconate (Sigma-Aldrich), 5 mM of KCl, 1 mM of MgCl_2_, 0.02 mM of CaCl_2_, 0.2 mM of EGTA (Sigma-Aldrich), 10 mM of HEPES (Sigma-Aldrich), 4 mM of Na_2_-ATP (Sigma-Aldrich), and 0.3 mM of Na-GTP (Sigma-Aldrich) (pH 7.3 with NaOH, 290–293 mOsm). In the current-clamp configuration, cells were held at −65 mV or −50 mV. During recording, access resistance was constantly monitored. Recordings were excluded if an input resistance changed over 15%. Clampex 10.7 software (Molecular Devices, San Jose, CA, USA) was used for data acquisition. The resting membrane potential (RMP) was measured from the resting-state membrane potential 1 min after the whole-cell patch-clamp was complete. The input resistance value was calculated from its steady-state membrane potential, and the voltage change was induced by hyperpolarizing current injection (−40 pA). The membrane time constant (membrane T) was obtained from recovery time for the membrane voltage to reach 63% of the steady-state voltage value from the hyperpolarizing current injection state (−40 pA). The first spike amplitude means the peak voltage value of the first spike in depolarizing current injection (20 pA), and the first spike latency represents the duration between the time point of the first spike peak value and the time point that start depolarizing current injection (20 pA). The firing frequency represented the firing numbers for 5 min, after 1 minuet from the whole-cell patch-clamp was performed. The action potential rise time was measured when the first spike reached 10% to 90% of the amplitude (peak relative to baseline). The action potential decay time constant (Decay T) was determined by fitting a falling phase of the spike to a single exponential function. The half-width of action potential was calculated by elapsing time when the voltage from the first spike cross half-maximal amplitude (peak relative to baseline) during the rising and the falling phase. 

### 4.5. Pharmacology Application

Ghrelin and leptin (mouse) were obtained from R&D Systems (Minneapolis, MN, USA). Drugs were diluted in ACSF to 100 nM before each experiment. In pharmacology application, the drug solutions were switched from normal ACSF for 1–2 min. During non-drug time, the recording chamber continuously flowed by normal ACSF. 

### 4.6. Data Analysis

All electrophysiological data were treated and expressed numerically using AxonTM pCLAMPTM10.1 Electrophysiology Data Acquisition & Analysis Software (Molecular Devices, San Jose, CA, USA). For the differences between individual neuron groups, *p* <  0.05 was interpreted as statistically significant (* *p*  <  0.05, ** *p*  <  0.005, *** *p*  <  0.001). All graphs were crafted in GraphPad^®^ Prism 7 (GraphPad Software Inc., La Jolla, CA, USA), and final arrangement and labeling were carried out using Adobe Illustrator CC 2019 (Adobe Inc., San Jose, CA, USA). All graph data are illustrated as mean ± standard error of the mean (SEM). Table 1 values are particularly treated as mean ± standard deviation (SD). The duration, such as half-width (or full width at half maximum, FWHM), was calculated by elapsing time when the voltage from the first spike crossed the half-maximal amplitude (peak relative to baseline) during the rising and the falling phase. The rising action potential time was measured when the first spike reached 10% to 90% of the amplitude (peak relative to baseline). The decay time constant of action potential was determined by fitting a falling phase of the spike to a single exponential function. 

## Figures and Tables

**Table 1:** Intrinsic properties of individual neurons.

Intrinsic Properties	DAT	AgRP	POMC
RMP (mV)	−52.24 ± 4.18 ^+^	−52.38 ± 3.80 ^#^	−60.54 ± 7.06 *^,+^
Input resistance (MΩ)	1286.88 ± 352.32 *	1751.73 ± 584.75 *^,#^	1321.86 ± 418.18 ^#^
Membrane τ (ms)	66.06 ± 22.45 *	87.22 ± 22.03 *^,#^	54.06 ± 18.43 ^#^
First spike amplitude (mV)	62.38 ± 16.68 ^+^	64.44 ± 12.16	73.48 ± 7.78^+^
First spike latency (ms)	81.80 ± 54.24 *	60.12 ± 18.36 ^#^	119.74 ± 53.73 *^,#^
Firing Frequency (Hz)	17.53 ± 7.03	19.56 ± 8.64 ^#^	13.57 ± 5.15 ^#^
Rising time (ms)	1.46 ± 0.59	1.83 ± 0.76	1.23 ± 0.60
Halfwidth (ms)	2.27 ± 0.42 ^+^	2.37 ± 0.38 ^#^	1.80 ± 0.32 ^#,+^
Decay τ (ms)	2.21 ± 0.69 ^+^	2.53 ± 0.50 ^#^	1.47 ± 0.35 ^#,+^
Firing pattern	burst	irregular burst	tonic
Firing mode	regular	adapting	adapting

Values are means ± SD of various measures. Each following symbol mean that they are signifi-cantly different *: DAT-AgRP ^#^: AgRP-POMC ^+^: DAT-POMC (*p* < 0.05, Tukey multiple comparison, one-way ANOVA test).

## Data Availability

All data reported in the manuscript.
